# Deleted in breast cancer-1 (DBC-1) in the interface between metabolism, aging and cancer

**DOI:** 10.1042/BSR20130062

**Published:** 2013-08-23

**Authors:** Eduardo Nunes Chini, Claudia C. S. Chini, Veronica Nin, Carlos Escande

**Affiliations:** *Laboratory of Signal Transduction, Department of Anesthesiology, Kodog Ageing Center, Mayo Clinic Cancer Center and Mayo Clinic College of Medicine, Rochester, MN 55905, U.S.A.

**Keywords:** aging, cancer, circadian cycle, deleted in breast cancer-1 (DBC1), epigenetics, histone deacetylase (HDAC), metabolism, ACC, acetyl-CoA carboxylase, AMPK, AMP-activated protein kinase, AR, androgen receptor, ATM, ataxia telangiectasia-mutated, ATR, ataxia telangiectasia and Rad3-related, CARP1, cell division cycle and apoptosis regulator protein 1, CCAR1, cell cycle and apoptosis regulator 1, DBC1, deleted in breast cancer-1, DBIRD, DBC1-ZIRD complex, ER, oestrogen receptor, ESA, essential for Sirt1 activity, GR, glucocorticoid receptor, HDAC, histone deacetylase, LZ, leucine zipper, mRNP, messenger ribonucleoprotein, NL, nuclear localization, PKA, protein kinase A, RAR, retinoic acid receptor, RNAPII, RNA polymerase II, TR, thyroid hormone receptor

## Abstract

DBC1 (deleted in breast cancer-1) is a nuclear protein that regulates cellular metabolism. Since alteration in cellular metabolism have been proposed to be the emerging ‘hallmark’ of cancer, it is possible that DBC1 may be implicated in the regulation of cancer cell energy metabolism. However, at this point any role of DBC1 in cancer is only speculative. In this review, we will discuss the new developments in DBC1 research, its molecular structure, regulatory roles and implication in metabolism, aging and cancer.

## INTRODUCTION

**Figure 1:**
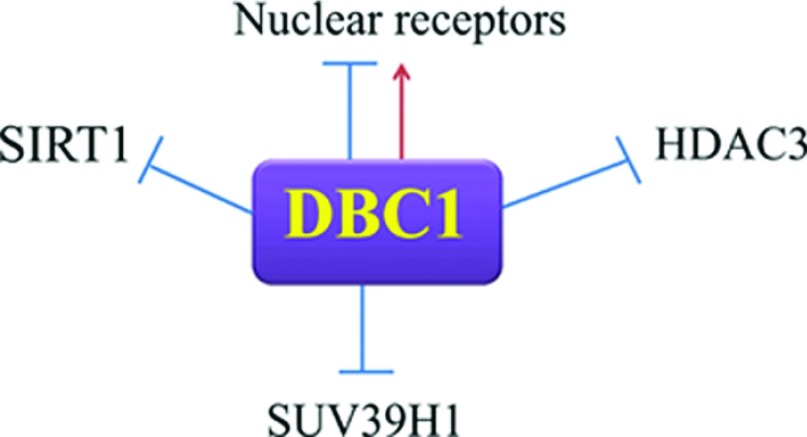
DBC1 a regulator of epigenetic modifiers and nuclear receptors DBC1 binds and regulates the function of several nuclear proteins and epigenetic modifiers such as nuclear receptors (e.g. AR, RAR and Rev-Erbα receptor), HDACs (SIRT1 and HDAC3), the methyltransferase SUV39H1.

DBC1 (deleted in breast cancer-1) is a nuclear protein that was originally proposed to be deleted in some breast cancers [1]. However, to date, no direct experimental evidence exists that implicates DBC1 on tumorigenesis [2–3]. The best characterized physiological roles of DBC1 are in the regulation of liver, fat metabolism and apoptosis [4–9]. Since alteration in cellular metabolism has been identified as an emerging ‘hallmark’ of cancer, it is possible that DBC1 may be implicated in the regulation of cancer cell energy metabolism. However, at this point any role of DBC1 in cancer metabolism is only speculative. Furthermore, DBC1 has clearly emerged as a nuclear receptor-binding protein, as it interacts with the ER (oestrogen receptor) and AR (androgen receptor) and the haem receptor Rev erb [10–15], as a regulator of epigenetic modifiers such as SIRT1, HDAC3 (histone deacetylase 3) and SUV39H1 [4–7,16,17] (Figure 1) and as a key component of the human spliceosome [28]. In this review, we will discuss the new developments in DBC1 research, its molecular structure, regulatory roles and implication in metabolism, aging and cancer.

## THE NAMESAKE CONFUSION

We should start this discussion with the unfortunate confusion that the current designated nomenclature for this protein may cause. DBC1 is not to be confused with another protein named deleted in bladder cancer-1 that receives the same abbreviation. In addition, DBC1 has received many other names and abbreviations including KIAA1967, P30 DBC protein, P30DBC1, NET35, DBC.1, P30 DBC2 3 and deleted in Breast Cancer Gene 1 Protein 3. These names in no way reflect any of the functional and structural characteristics of this protein. Here we will use the abbreviation DBC1 to refer to this protein. However, it is clear that a more appropriate and descriptive nomenclature is necessary for this protein. To further complicate the nomenclature DBC1 is a paralogue of the protein named CCAR1 (cell division cycle and apoptosis regulator protein 1) [27].

## IS DBC1 REALLY DELETED IN CANCERS?

The human *DBC1* gene is localized to 8p22, a region that was previously described to be homozygously deleted in some breast cancers [1]. It was therefore postulated that *DBC1* is a gene deleted in cancers that could have an important role in the development and progression of tumours [1–3]. However, to date no direct data exist to implicate *DBC1* with any aspect of tumorigenesis, and whether *DBC1* is a tumour suppressor or a tumour promoter is the subject of intense speculation [1–3,18–26].

In contrast to the initial report that DBC1 was deleted in breast cancers, some recent studies fail to observe deletion of DBC1 in several types of cancer cells in culture or in tumour tissues including breast, gastric, oesophageal, pancreatic and others [1–3,18–26]. Furthermore, to date the role of DBC1 in the pathogenesis of cancer is only hypothetical. Several correlational studies have attempted to implicate DBC1 on the pathogenesis of cancers [18–26]. However, the causal relationship between loss of DBC1 and tumorigenesis has not been established.

Nonetheless, based on its many cellular functions, the potential role of DBC1 in the regulation of metabolism, aging and tumour biology is of great importance and needs to be further explored. Interestingly, DBC1 is a regulator of several molecules and pathways that have been implicated in the pathogenesis of cancer such as apoptosis, nuclear receptor function, cellular metabolism, circadian cycle and epigenetics [4–17].

## STRUCTURE AND FUNCTIONAL ORGANIZATION OF DBC1

**Figure 2:**
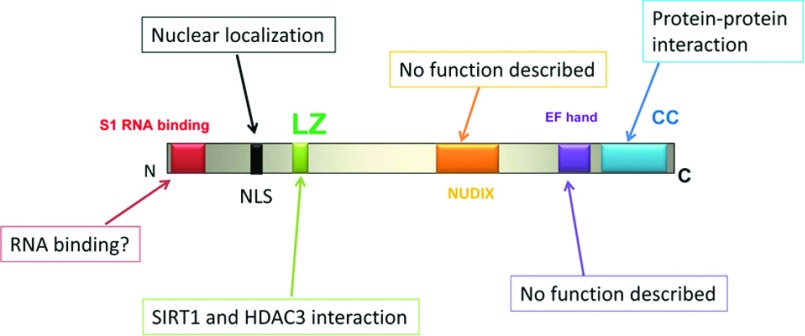
Functional structure of DBC1 The Figure describes the domains of DBC1 and its probable functions. Abbreviations N, N-terminus; C, C-terminus; NLS, nuclear localization sequence; LZ, leucine zipper; nudix, **Nu**cleoside **Di**phosphate linked to **X;** EF-hand, (where EF stands for the E and F alpha helices of parvalbumin) are Ca^2+^-binding domains present in different Ca^2+^-binding proteins); CC, coiled coil.

As described above DBC1 is a paralogue of the protein CCAR1 [27]. Both these proteins are large multi-domain proteins, with a predominantly NL (nuclear localization) [27]. Comparative studies of the structure of these two proteins have led to the description of several important domains in DBC1 [27]. Both DBC1 and CCAR1 share a NL motif, a CC (coiled-coil) domain that is important for the formation of protein–protein interaction, an inactive EF-hand that is unlikely to bind Ca^2+^ ions, a Nudix domain, a RNA-binding domain and an LZ (leucine zipper) (Figure 2). The role of these domains is discussed in detail below.

### The Nudix domain

The Nudix hydrolase family is a group of enzymes that have great substrate muiltispecificity and ambiguity [27]. Some catalyse the degradation of several nucleotides such as ADP-ribose or Ap4A (diadenosine tetraphosphate) [27], and effectively remove pyrophosphate from the 5′ region of mRNA [27]. The Nudix domain of DBC1 is likely to be catalytically non-functional [27], because of loss of key acidic residues in the active site motif. However, the DBC1 Nudix domain has been postulated to bind nucleotides and nucleotide binding to DBC1 could regulate its functions [27]. In fact, a similar non-functional Nudix domain has been shown to play a role on the regulation of calcium channels by ADP-ribose [27]. However, to date no studies have shown any functional role for the Nudix domain on DBC1.

### The RNA-binding domain and DBC1 role in the spliceosome

Another interesting domain present in DBC1 is the N-terminal S1-like RNA-binding domain [27]. The presence of this RNA-binding domain is consistent with the role of DBC1 on the splicing of RNA [27,28]. Recently, it has been described that DBC1 and the protein ZNF326 form a complex with the RNAPII (RNA polymerase II), and assemble it into an mRNP (messenger ribonucleoprotein) [28]. This DBC1–ZNF326 complex was named the DBIRD (DBC1–ZIRD complex) [28]. The DBIRD complex functions at the interface between core mRNP particles and RNAPII, affecting local transcript elongation rates and alternative splicing; it is possible that the RNA-binding domain of DBC1 may have a key role on this function. However, again the actual role of this domain in the spliceosome function has not been investigated.

### The N-terminal region regulates epigenetic modifiers and nuclear receptors

The N-terminal region of DBC1 is required for binding to the deacetylases SIRT1 and HADC3, and the methyltransferase SUV39H1. Within the N-terminal domain, the LZ region is critical for the binding of HDAC3; however, there is contradictory evidence about the importance of the LZ domain for the interaction with SIRT1 [6,17]. The first 240 amino acids of DBC1, a region that does not contain the LZ, is responsible for the interaction with SUV39H1. [2,4,6,16,17]. DBC1 binds directly to the catalytic site of these enzymes, inhibiting their activities [4,6,16,17]. The interaction between DBC1 and these binding partners appear to be direct and may not require additional cofactors, since it can be detected both in cell and *in vitro* using recombinant purified proteins [4,6,16,17]. The specific region of DBC1 necessary for the interaction with nuclear receptors varies, but in many cases depends on its N-terminus.

## THE MULTIPLE INTERACTIONS OF DBC1

Owing to its multiple domains, DBC1 has been shown to have many protein–protein interactions and to regulate several cellular processes. DBC1 is a regulator of nuclear receptors such as ERα and ERβ and the AR, the RAR (retinoic acid receptor) and of the haem receptor Rev-erbα [10–15]. DBC1 binds to these receptors and modulates their transcriptional activity and/or stability. In addition to modulating transcriptional activity of nuclear receptors, we [4,16] and others [5–7,17] have shown that DBC1 regulates epigenetic modifiers such as the deacetylases HDAC3 and SIRT1, and the methyl-transferase SUV39H1. Owing to these multiple protein–protein interactions DBC1 appears to have multiple cellular functions including regulation of apoptosis, metabolism, browning of adipocyte, RNA splicing and circadian cycle [4–9,16,28].

### DBC1 is a regulator of nuclear receptors

As described above, DBC1 interacts and regulates the stability and function of several nuclear receptors including the ER, the AR, the retinoic receptor and the circadian cycle receptor Rev-erbα [10–15]. The effect of DBC1 appears to be different depending on the receptor that it interacts with, although in general it appears to function as a co-activator. For example, DBC1 has been shown to be a ligand dependent co-activator for the ERα, the GR (glucocorticoid receptor) and the TR (thyroid hormone receptor) [14]. Interestingly, DBC1 also binds to the CCAR1 (cell cycle and apoptosis regulator 1), an important co-activator for several nuclear receptors. DBC1 and CCAR1 synergistically enhance transcription activation mediated by the ERα, TR and GR. DBC1 is also part of the co-activator protein complex of the AR and promotes AR DNA binding [11]. DBC1 was also reported to be required for transcriptional activity of the RARα [10]. In contrast, DBC1 functions as a repressor of the transcriptional activity of the ERβ [12], and BRCA1 (breast cancer early-onset 1) [21], suggesting that DBC1 could be a more general regulator of transcription.

### The interaction between DBC1 and nuclear receptor is both ligand-dependent and ligand-independent

Binding of DBC1 to AR is ligand-dependent, involves the N-terminal region of DBC1, and does not significantly affect AR stability [11]. The ERα also binds to the N-terminal domain of DBC1, but there are contradictory data on whether DBC1 regulates ERα protein stability [13,14]. Although in an earlier report DBC1 was shown to regulate the steady-state level of unliganded protein [13], a more recent study found that DBC1 did not affect the levels of this receptor and that it binds to ERα both in the presence and absence of ligand [14].

### DBC1 interacts and regulates the circadian cycle modulator Rev-erbα

Interestingly DBC1 interacts with and regulates the circadian receptor Rev-erbα receptor [29,30]. Rev-erbα is a haem receptor that coordinates the circadian regulation of cell metabolism, and clock genes [29–33]. Loss of Rev-erbα led to hepatosteatosis, and circadian cycle dysregulation [34–36]. These effects are more pronounced in mice lacking both Rev-erbα and Rev-erbβ [35]. These findings establish that Rev-erb regulates several circadian and metabolic processes [29,30].

A discussed above, the interaction between DBC1 and proteins such as nuclear receptors and the deacetylases SIRT1 and HDAC3 occur through the N-terminal region of DBC1 and it mostly depends on its LZ domain [4–6,16]. Surprisingly, the interaction between DBC1 and Rev-erbα is dependent on the CTD (C-terminal domain) of DBC1 and not on the N-terminal or the LZ domain of DBC1 [15]. These data indicate that through its different domains, DBC1 may interact with multiple partners at the same time. Furthermore, both the C- and N-terminal regions of DBC1 were necessary for the proper function and stabilization of the Rev-erbα receptor [15]. As described for other nuclear receptors the binding of DBC1 to Rev-erbα is at least in part modulated by ligands [15].

Elucidating the connections between DBC1, HDAC3 and Rev-erbs may have implications for the pathogenesis and treatment of metabolic diseases like obesity, diabetes, liver steatosis, metabolic syndrome and cancer. The recent development of Rev-erb agonists that alter circadian behaviour, decrease obesity and adipogenesis [37] suggests that these pathways can be targeted to improve circadian rhythm and metabolism [37]. In this regard, it is possible that targeting DBC1-Rev-erbα interaction may have important implications for the treatment of metabolic diseases and cancer. In fact, we described that DBC1 was required for the cellular circadian oscillations of Rev-erbα, Bmal1 and SIRT1 activity suggesting that DBC1 is a key regulator of the circadian rhythm [15].

## DBC1 IS AN ENDOGENOUS REGULATOR OF THE EPIGENETIC MODIFIERS SIRT1, HDAC3 AND THE METHYLTRANSFERASE SUV39H1

The recent discovery of DBC1 as an endogenous inhibitor of SIRT1, HDAC3 and the methyltransferase SUV39H1 [4–7,16,17] suggests that DBC1 plays a crucial physiological role as a modulator of epigenetics and metabolic function. Of particular interest is the key role of both SIRT1 and other HDACs as regulators of metabolic functions and gene networks [38–45].

### DBC1 is a key regulator of SIRT1

The NAD^+^-dependent deacetylase SIRT1 is a metabolic master switch that is part of several physiological pathways [45]. The discovery of SIRT1 has been one of the most exciting recent advances in the study of energy metabolism and aging [45]. SIRT1 uses NAD^+^ as a substrate to promote deacetylation of several target proteins [38,39]. These changes in protein acetylation have an important impact on several cellular processes [38,39,45]. SIRT1 activation delays some features of aging and protects against liver steatosis, type II diabetes and cancer [38,39,43–45]. Although controversial, it appears that SIRT1 may also mediate some of the beneficial effects of caloric restriction [38,39,45]. Interestingly, both caloric restriction and aging have important consequences on metabolism and cancer biology [38,39,45]. It appears that caloric restriction protects and aging increases the susceptibility to several metabolic dysfunctions and cancers. [38,39,45]. These observations indicate that the regulation of SIRT1 by DBC1 may influence energy metabolism, aging and cancer cell biology [38,39,43–45].

**Figure 3:**
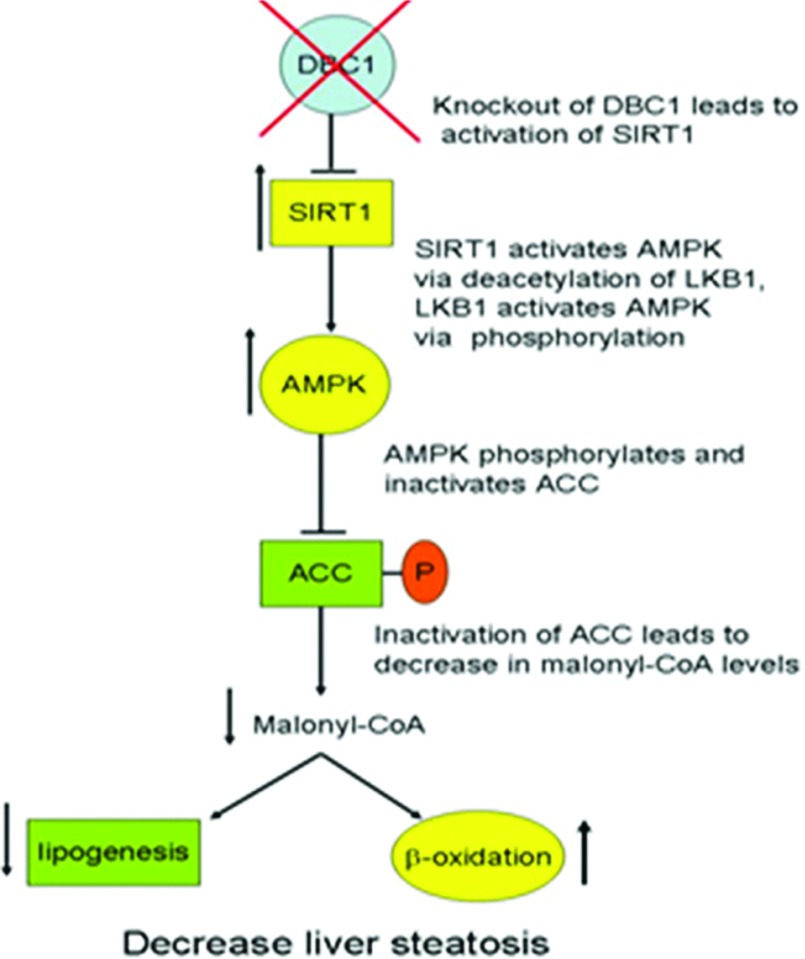
Regulation of liver metabolism by DBC1 We have shown that DBC1 plays a crucial role in the development of liver steatosis by direct binding and inhibiting SIRT1, with the subsequent modulation of downstream effects of SIRT1 on hepatic lipogenesis and β-oxidation via AMPK [4]. It is likely that knockout of DBC1 decreases fatty liver infiltration by inducing SIRT1/LKB1-dependent activation of AMPK. AMPK phosphorylates and inactivates ACC. Inactivation of ACC leads to subsequent decrease in malonyl-CoA. This decrease in malonyl-CoA levels increases hepatic β-oxidation and reduced lipogenesis, preventing the development of liver steatosis.

**Figure 4:**
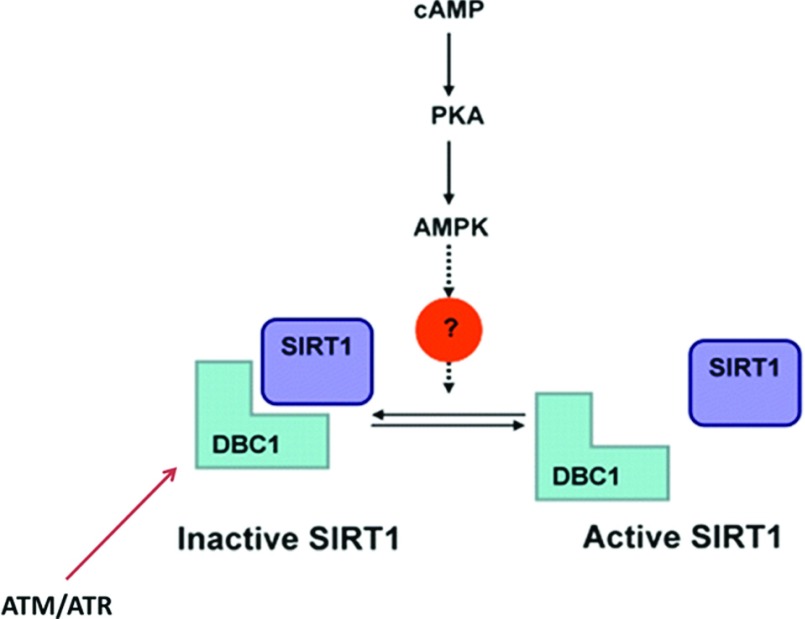
Regulation of the SIRT1–DBC1 interaction by signalling pathways The SIRT1–DBC1 interaction is regulated by metabolic inputs and also by the cAMP–PKA, the AMPK and the ATM/ATR pathways.

We and others have demonstrated that DBC1 is a key physiological regulator of SIRT1 activity [4–7]. We have previously described that the activation of SIRT1 during fasting is mediated by the dissociation of the SIRT1–DBC1 complex [4]. Furthermore, we observed that deletion of DBC1 in mice leads to SIRT1 activation and the protection against the development of some features of metabolic syndrome such as liver steatosis (Figure 3) [4]. We believe that these effects are mediated by a cascade of events that finalize in the activation of SIRT1 and the AMPK (AMP-activated protein kinase) pathway (Figure 4) [46]. In fact, we have characterized the effects of DBC1 downstream of SIRT1, such as the regulation of AMPK activity, and its effects on lipogenesis, and β-oxidation, particularly malonyl-CoA synthesis by ACC (acetyl-CoA carboxylase) (Figure 3) [4,46]. Deletion of DBC1 also promotes the expression of brown fat tissue genes in the white fat depots during cold exposure, a process known as ‘browning’ of the white fat. This is probably mediated by a SIRT1-dependent deacetylation of PPARγ (peroxisome-proliferator-activated receptor γ) [5]. DBC1 knockout mice also become glucose intolerant as they age, although the reason for this is still unknown [5].

As discussed, SIRT1 interacts with the LZ domain of DBC1 [4,6], and DBC1 appears to bind to the catalytic site of SIRT1, inhibiting it [4,6]. However, the mechanism of modulation of SIRT1 activity by DBC1 appears to be more complex. Recently, it was proposed that a 25 amino-acid region in the C-terminus of SIRT1 named the ESA (essential for Sirt1 activity) region functions as an ‘on switch’ for the deacetylase core of Sirt1 [47], and that the LZ region of DBC1 appears to inhibit Sirt1 by competing with the ESA region for a binding site in the catalytic domain [47]. In this regard, it appears that DBC1 does not simply block the catalytic site of SIRT1, but modulates the intramolecular interaction between the ESA and the catalytic site of SIRT1 necessary for deacetylase activation [47]. Independent of the precise molecular mechanism of modulation of SIRT1 activity by DBC1, and important aspect of this regulation is that it is targeted by metabolic and signalling pathways.

Indeed, the SIRT1–DBC1 interaction is regulated by inputs from several signalling pathways and also the metabolic state of the cell [4,46,48–52]. This observation indicates that the SIRT1–DBC1 interaction may serve as a metabolic sensor. We have previously observed that high caloric loads increase the interaction between SIRT1 and DBC1 leading to SIRT1 inhibition [4)] In contrast, fasting leads to a decrease in SIRT1–DBC1 interaction and activation of SIRT1 (Figure 4). To understand the mechanisms that are used by cells to sense metabolic changes, is of major importance to characterize the role of DBC1 in the regulation of SIRT1 activity during different caloric loads, and to determine the molecular mechanisms that regulate the DBC1–SIRT1 interaction. Of major interest is the observation that the SIRT1–DBC1 interaction is modulated by input from the cAMP–PKA (protein kinase A) and AMPK signalling pathways [46]. Both these pathways activate SIRT1 by modulating the SIRT1–DBC1 interaction (Figure 4). In addition, it has also been described that the SIRT1–DBC1 interaction can be regulated by a DNA damage response pathway [50,51]. Upon DNA damage the ATM (ataxia telangiectasia-mutated)/ATR (ataxia telangiectasia and Rad3-related) cascade is activated, and DBC1 is phosphorylated at Thr^454^ by these kinases. The phosphorylation of DBC1 increases its interaction with SIRT1, and inhibits SIRT1 [50,51]. It is therefore clear that the SIRT1–DBC1 interaction is dynamic and is regulated by inputs from different signalling pathways.

### DBC1 is a regulator of HDAC3

Although much attention has been focused on the regulation of SIRT1 by DBC1, we postulated that DBC1 could regulate other deacetylases [16]. In fact, one of the deacetylases that shares similar substrates and physiological roles with SIRT1 is the member of the class I family of deacetylases HDAC3. The members of this class are ubiquitously expressed, but unlike HDAC1 and HDAC2, which are nuclear proteins, HDAC3 can be found in both the nuclei and cytoplasm of cells. Similar to other class I HDACs, HDAC3 represses transcription when directed to promoter regions by serving as a co-repressor [53]. Interestingly, SIRT1 and HDAC3 share several substrates such as p53, MEF2D (myocyte enhancer factor 2D) and NF-κB (nuclear factor κB), and both associate with p300/CBP (cAMP-responsive element-binding protein-binding protein) acetyltransferase. Furthermore, both SIRT1 and HDAC3 have been implicated as regulators of several common cellular and physiological functions such as apoptosis, circadian cycle, glucose and lipid metabolism and cancer [30,33,42,53,54]. Based on these observations we proposed that HDAC3 and SIRT1 could share not only substrates, but also regulatory proteins such as DBC1 [16] (Figure 1). In fact, we observed that DBC1 and HDAC3 interact and that DBC1 modulates HDAC3 activity [16]. In the absence of DBC1, HDAC3 activity is increased [16]. Furthermore, the HDAC3–DBC1 interaction may also modulate the cellular localization of HDAC3 [16]. The mechanisms that modulate the HDAC3–DBC1 interaction have not been explored yet, but it is likely that akin to SIRT1 the interaction of DBC1 with HDAC3 may also be modulated by signalling pathways and metabolic inputs.

### DBC1 regulates the methyltransferase SUV39H1

Finally, DBC1 has also been shown to interact and regulate the methyl-transferase SUV39H1. SUV39H1 is a histone H3K9-specific methyltransferase important for heterochromatin formation. DBC1 directly binds to the SUV39H1 catalytic domain and inhibits its ability to methylate histone H3 [17] (Figure 1). Because both SIRT1 and SUV39H1 bind to the N-terminal domain of DBC1, a trimeric complex is not possible. In fact, it was reported that binding of SUV39H1 to DBC1 dissociates the SIRT1-SUV39H1 complex [17]. These data combined indicates that DBC1 regulates several components of the epigenetic machinery, modulating the function and activity of writers and erasers [4–7,16,17]. Elucidating the precise role of DBC1 in epigenetics and its implications for the regulation of metabolism, aging and cancer will probably be an important addition to our present knowledge of cell biology. Furthermore, we speculate that the list of epigenetic modifiers regulate by DBC1 may increase in the future.

## CONCLUSIONS

In recent years, several metabolic sensors and metabolic regulated signalling pathways have been uncovered. Of particular interest are the findings that indicate that cellular metabolism has a key impact on the regulation of gene networks [30–34,38–45,48,55,56]. In fact, epigenetic modulation is one of the mechanisms that appear to receive a large amount of input from metabolic pathways [30–34,38–45,48,55,56]. Epigenetic changes such as histone acetylation and DNA methylation are a powerful mechanism of modulation of gene function that play a role in the development of many human conditions such as cancer, diabetes, obesity and aging [38–40,45,56]. Epigenetic modifications can either activate on inactivate genes and are tightly modulated by metabolic inputs [38–45]. Of these epigenetic modifications protein lysine acetylation has been shown to be modulated by metabolic pathways both via the acetyltransferase and deacetylases [38–45]. The discovery of a family of NAD^+^-dependent deacetylases that promote protein lysine deacetylation has greatly advanced our understanding of the interaction between metabolism, epigenetic changes and signalling pathways [38–40,45,56]. These enzymes, the Sirtuins, are localized in several cellular compartments and are key regulators of energy metabolism, gene function and also interface with several key cellular signalling pathways such as the cAMP–PKA, AMPK and mTOR (mammalian target of rapamycin) pathway [38,39,45]. The effect of Sirtuins on cellular function expands way beyond histone deacetylation [38,39,45,55,57]. The number of proteins that are deacetylated by Sirtiuns and the interconnection between these enzymes with cellular metabolism and signalling pathways continues to expand [38,39,45,55,57]. In addition, to the sirtuins, other HDACs such as HDAC3 have been implicated in the regulation of cellular metabolism and epigenetic changes [30,33]. Of particular interest is the fact that these deacetylases also appear to be modulated and interact with several nuclear proteins including nuclear receptors [30,33,55,57]. In fact, several nuclear receptors have a role on the regulation of metabolism and epigenetic [56]. The discovery of DBC1 as a modulator of SIRT1, HDAC3, SUV39H1 and several nuclear receptors raises the possibility that DBC1 may be a master regulator of the interconnection between metabolism, and epigenetics. In this regard these roles of DBC1 may have extreme importance for metabolic diseases, aging and cancer cell biology (Figure 1). The study of this multifunctional protein is likely to provide novel understanding and potential new therapeutic options for multiple human diseases and conditions such as obesity, metabolic syndrome, atherosclerosis, inflammation, aging and cancer.
